# Systematic review of HIV treatment adherence research among people who inject drugs in the United States and Canada: evidence to inform pre-exposure prophylaxis (PrEP) adherence interventions

**DOI:** 10.1186/s12889-018-6314-8

**Published:** 2019-01-08

**Authors:** Angela R. Bazzi, Mari-Lynn Drainoni, Dea L. Biancarelli, Joshua J. Hartman, Matthew J. Mimiaga, Kenneth H. Mayer, Katie B. Biello

**Affiliations:** 1 Department of Community Health Sciences, Boston University School of Public Health, Boston, MA USA; 2 Department of Health Law, Policy & Management, Boston University School of Public Health, Boston, MA USA; 3 Section of Infectious Diseases, Department of Medicine, Boston University School of Medicine, Boston, MA USA; 4 Evans Center for Implementation and Improvement Sciences, Boston University School of Medicine, Boston, MA USA; 5 Center for Healthcare Organization and Implementation Research, Edith Nourse Rogers Memorial Veterans Hospital, Bedford, MA USA; 6 Boston Medical Center, Boston, MA USA; 7 Departments of Behavioral & Social Sciences and Epidemiology, Center for Health Equity Research, Brown University School of Public Health, Box G-S121-8, Providence, RI 02912 USA; 8 Department of Psychiatry & Human Behavior, Brown University Alpert Medical School, Providence, RI USA; 9 Center for Health Equity Research, Brown University, Providence, RI USA; 10 The Fenway Institute, Fenway Health, Boston, MA USA; 11 Department of Medicine, Beth Israel Deaconess Medical Center and Harvard Medical School, Boston, MA USA

**Keywords:** Antiretroviral therapy, Pre-exposure prophylaxis, People who inject drugs, Drug use, Adherence, Prevention

## Abstract

**Background:**

People who inject drugs (PWID) are at increased risk for HIV acquisition and could benefit from antiretroviral pre-exposure prophylaxis (PrEP). However, PrEP has been underutilized in this population, and PrEP adherence intervention needs are understudied.

**Methods:**

To inform PrEP intervention development, we reviewed evidence on antiretroviral therapy (ART) adherence among HIV-infected PWID. Guided by a behavioral model of healthcare utilization and using the PICOS framework, we conducted a systematic review in four electronic databases to identify original research studies of ART adherence in HIV-infected PWID in the United States and Canada between Jan 1, 2006–Dec 31, 2016. We synthesized and interpreted findings related to developing recommendations for PrEP adherence interventions for PWID.

**Results:**

After excluding 618 duplicates and screening 1049 unique records, we retained 20 studies of PWID (mean *n* = 465) with adherence-related outcomes (via pharmacy records: *n* = 9; self-report: *n* = 8; biological markers: *n* = 5; electronic monitoring: *n* = 2). Predisposing factors (patient-level barriers to adherence) included younger age, female sex, and structural vulnerability (e.g., incarceration, homelessness). Enabling resources (i.e., facilitators) that could be leveraged or promoted by interventions included self-efficacy, substance use treatment, and high-quality patient-provider relationships. Competing needs that require specific intervention strategies or adaptations included markers of poor physical health, mental health comorbidities (e.g., depression), and engagement in transactional sex.

**Conclusions:**

HIV treatment adherence research carries important lessons for efforts to optimize PrEP adherence among PWID. Despite limitations, this systematic review suggests that strategies are needed to engage highly vulnerable and marginalized sub-groups of this underserved population (e.g., younger PWID, women who inject drugs) in PrEP adherence-related research and programming.

**Electronic supplementary material:**

The online version of this article (10.1186/s12889-018-6314-8) contains supplementary material, which is available to authorized users.

## Background

Injection drug use contributes to approximately 10% of new HIV infections globally and 30% outside of Africa [1]. Although syringe exchange programs help reduce HIV acquisition among people who inject drugs (PWID) [2], access is insufficient in some locations, and sexual and injection-related HIV risk behaviors persist in many PWID populations. For example, in the United States, where PWID accounted for 9% of new HIV infections in 2015, only 34% of HIV-uninfected PWID received all their syringes from sterile sources and 72% reported past-year condomless sex or receptive syringe sharing [3]. As evidenced by recent HIV outbreaks linked to injection drug use (e.g., in Indiana, 2015) [4] and elevated incidence of hepatitis C virus (HCV), considered a harbinger of HIV outbreaks [5], the introduction of HIV into PWID networks could reverse decades of HIV prevention success.

Antiretroviral pre-exposure prophylaxis (PrEP) is efficacious in preventing HIV acquisition among PWID and its provision has been recommended for high risk PWID (along with other essential health and harm reduction services) by the World Health Organization [6] and an increasing number of national health departments [7, 8]. In the only clinical trial conducted among PWID to date, the Bangkok Tenofovir Study, daily oral PrEP (tenofovir disoproxil fumarate) resulted in a 48.9% reduction in HIV incidence (95% CI, 9.6–72.2; *p* = 0.01) [9]. Similar to other clinical trial results [10], efficacy increased with adherence to the daily oral regimen, reaching 58.0% among PWID reporting ≥75% adherence and 83.5% with ≥97.5% adherence [11]. Suboptimal adherence was associated with being male, being younger (< 40 years), and reporting recent methamphetamine injection, incarceration, or sex with casual partners [11]. Among participants in the open-label extension of this clinical trial, three quarters self-reported taking less than 90% of daily PrEP doses [12]. While these data suggest that taking PrEP daily could be challenging for PWID, little is known about real-world challenges with PrEP adherence among PWID because uptake in this population has been low [13]. With efforts to improve PrEP access and delivery to PWID currently underway [14], understanding adherence to medications in this population, defined here as the process by which patients take medications as prescribed (including initiation, implementation, and discontinuation) [15], could help inform future research and interventions to promote PrEP adherence.

Due to the paucity of research on PrEP adherence among PWID, we sought to review the literature on adherence to antiretroviral treatment (ART) medications in HIV-infected PWID for several reasons. First, both ART and PrEP, which are currently formulated and approved as daily medications, require adherence to be effective. Second, syndemic substance use, related mental health comorbidities, and structural and socioeconomic vulnerabilities that are known to adversely impact ART adherence among PWID [16–18] would likely also pose challenges to PrEP adherence. For example, HIV-uninfected PWID likely experience many of the same medical and psychiatric comorbidities, socioeconomic vulnerabilities, and challenges to healthcare utilization experienced by HIV-infected PWID [19]. Indeed, interventions to optimize ART adherence have been successfully adapted to support PrEP adherence in other populations such as men who have sex with men [20]. It is thus likely that a comprehensive understanding of the factors that influence ART adherence among HIV-infected PWID could help inform interventions to promote PrEP adherence among HIV-uninfected PWID. To this end, we conducted a systematic review following the PICOS framework to synthesize evidence in response to the research question: *“What is known about ART adherence among PWID that could inform PrEP adherence interventions for this population?”*

## Methods

To guide our review of existing evidence on ART adherence among PWID, we drew from the Behavioral Model of Healthcare Utilization for Vulnerable Populations [21]. In this model, *predisposing factors* are factors that could directly impact adherence: socio-demographics, substance use barriers, and related sources of social and structural vulnerability. *Enabling factors* are resources that could facilitate PrEP adherence and be targeted by interventions, such as individual resources and health service facilitators (e.g., characteristics of healthcare providers and services). *Need-related factors* include health status barriers, beliefs and perceptions, and health risks that could also influence adherence. Identifying challenges within all of these domains helps highlight specific intervention targets, adaptations, or referral systems.

### Reporting rationale

Reporting of this study is in accordance with PRISMA guidelines and PICOS framework. The protocol has been registered and detailed methods are described herein.

### Search strategy and selection criteria

We followed systematic and scoping review methodologies [22, 23] to address the overarching research question, *“What is known about ART adherence among PWID that could inform PrEP adherence interventions for this population?”* Original research studies were eligible if they were published in English between January 1, 2006–December 31, 2016; included HIV-infected, non-institutionalized U.S. and Canadian samples with the majority (> 50%) reporting past 6-month injection drug use; i.e., the population); assessed and/or tried to improve ART adherence (i.e., the intervention); and reported specific ART adherence-related outcomes including implementation or the extent to which actual dosing corresponded to prescribed regimens (i.e., the outcome). Based on our overarching goal of informing PrEP adherence interventions, we limited our review to the United States and Canada because PrEP had been approved and original research studies on ART adherence among PWID were available. While we aimed to systematically assess the highest quality of evidence, a lack of studies using experimental designs forced us to include all study designs, including randomized controlled trials, prospective cohort studies, cross-sectional surveys and qualitative research (i.e., study design). To identify eligible studies, relevant search terms were applied in PubMed, Web of Science, PsycINFO, and EMBASE (Additional file 1).

### Review process

After removing duplicate records, two independent reviewers screened titles and abstracts for inclusion criteria. Review of reference lists and natural language searches identified 3 additional eligible studies. Reviewers read full texts to confirm eligibility and extract data (e.g., number of participants) and findings (e.g., proportions, odds ratios significant in bivariate or multivariable analyses at the 10% level). We used a narrative synthesis approach [24] to assess, summarize and interpret evidence in relation to the domains of our conceptual model, evidence gaps, and implications for PrEP adherence intervention development. Rather than rank the quality of each study, we assessed the relevance of the findings for PrEP adherence and intervention development with PWID and described limitations of the studies included.

## Results

**Fig. 1:**
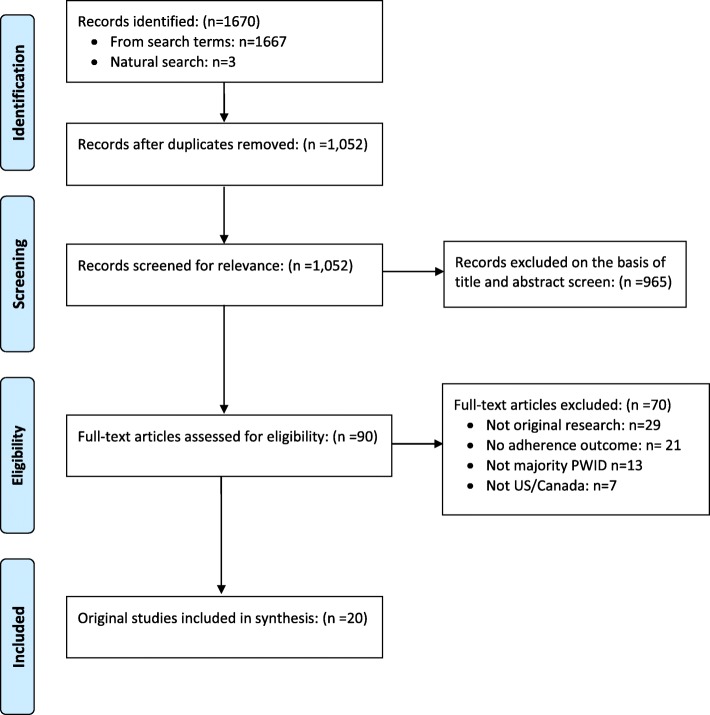
Flowchart of study identification and screening for inclusion in systematic review

Our search identified 1670 citations. After excluding 618 duplicates, we screened titles and abstracts of 1052 unique records and excluded 965 studies (Fig. 1). Review of 90 full texts resulted in exclusion of 70 studies (not original research studies: *n* = 29; no adherence outcomes: *n* = 21; not majority PWID: *n* = 13; not U.S./Canada: *n* = 7), resulting in a final sample of 20 studies (Table 1). Average sample size was 465 (standard deviation: 269; range: 57–966). All studies employed quantitative methods (longitudinal: *n* = 15, cross-sectional: *n* = 5). ART adherence outcomes were assessed using pharmacy records (prescription refill data; *n* = 9), self-report (*n* = 8), biological markers (viral load; *n* = 5), and electronic monitoring (*n* = 2). Studies identified the following predisposing, enabling, and need-related correlates of ART adherence (Table 2).

**Table 1:** Studies of ART adherence among people who inject drugs included in systematic review

Author, Year	Site(s), Sample	Study Design	Objective(s)	Outcome(s)
Altice et al., 2007 [49]	New Haven, CT (*n* = 141)^a^	Randomized controlled trial	Effect of DAART intervention on HIV RNA and CD4 count	Viral load suppression, change in CD4 count, and self-reported adherence
Arnsten et al., 2007 [46]	Baltimore, MD, Miami, FL, New York, NY, San Francisco, CA (*n* = 636)^b^	Cross-sectional survey	Factors associated with ART adherence and medication errors	Self-reported adherence
Azar et al., 2015 [42]	Vancouver, BC (*n* = 692)^c^	Prospective cohort	Effect of illicit drug use patterns on ART adherence	Pharmacy records (prescription refill data)
Bach et al., 2015 [52]	Vancouver, BC (*n* = 794)^d^	Prospective cohort	Effect of methadone discontinuation on ART adherence	Pharmacy records (prescription refill data)
Gonzalez et al., 2013 [56]	Boston, MA (*n* = 121)	Cross-sectional survey	Association of substance use with ART adherence	Electronic monitoring (MEMS cap data)
Hadland et al., 2012 [25]	Vancouver, BC (*n* = 545)^c^	Prospective cohort	Effect of age on ART adherence and viral load suppression	Pharmacy records (prescription refill data), viral load suppression
Ing et al., 2013 [44]	New Haven, CT (*n* = 74)^a^	Prospective cohort within a randomized controlled trial	Patterns of medication non-persistence (in DAART intervention group)	Electronic monitoring (MEMS cap data), viral load suppression
Joseph et al., 2015 [29]	Vancouver, BC (*n* = 703)^d^	Prospective cohort	Predictors of ART adherence	Pharmacy records (prescription refill data)
Kang et al., 2011 [26]	New York, NY, Bayamón, PR (*n* = 260)	Cross-sectional survey	Association of gender with HIV care	Self-reported use of HIV medications
Kavasery et al., 2009 [31]	Baltimore, MD (*n* = 335)^e^	Prospective cohort	Predictors of ART use and non-use	Self-reported dates of ART interruption and initiation
Knowlton et al., 2006 [33]	Baltimore, MD, Miami, FL, New York, NY, San Francisco, CA (*n* = 466)^b^	Cross-sectional survey	Factors associated with undetectable viral load	Viral load suppression
Knowlton et al., 2010 [34]	Baltimore, MD, Miami, FL, New York, NY, San Francisco, CA (*n* = 703)^b^	Prospective cohort	Predictors of ART use	Self-reported adherence
Palepu et al., 2006 [32]	Vancouver, BC (*n* = 278)^c^	Prospective cohort	Effect of methadone therapy on ART adherence, viral load suppression, and CD4 counts	Pharmacy records (prescription refill data), viral load suppression, change in CD4 count
Palepu et al., 2011 [28]	Vancouver, BC (*n* = 545)^d^	Prospective cohort	Effect of homelessness on ART adherence	Pharmacy records (prescription refill data)
Purcell et al., 2007 [50]	Baltimore, MD, Miami, FL, New York, NY, San Francisco, CA (*n* = 966)^b^	Randomized controlled trial	Effect of behavioral intervention on HIV transmission behavior, utilization of primary care, and ART adherence	Self-reported sexual and injection behaviors, self-reported number of primary care visits, self-reported adherence
Reddon et al., 2014 [53]	Vancouver, BC (*n* = 408)^d^	Prospective cohort	Effect of methadone therapy on ART discontinuation	Prescription refill data
Tapp et al., 2011 [27]	Vancouver, BC (*n* = 545)^d^	Prospective cohort	Effect of gender on ART adherence	Prescription refill data
Uhlmann et al., 2010 [54]	Vancouver, BC (*n* = 231)^d^	Prospective cohort	Effect of methadone therapy on ART initiation and adherence	Time to first ART use from pharmacy records (prescription refill data)
Waldrop-Valverde et al., 2008 [51]	South Florida, FL (*n* = 57)	Cross-sectional survey	Associations of low literacy and cognitive impairment with ART adherence	Self-reported adherence
Westergaard et al., 2013 [30]	Baltimore, MD (*n* = 790)^e^	Prospective cohort	Predictors of linkage to and retention in HIV care, and viral load suppression	Self-reported appointment attendance, self-reported lapses in care, viral load suppression

^a^DAART intervention study in New Haven, CT (ClinicalTrials.gov identifier NCT00367172; *n* = 2)

^b^INSPIRE study; multisite including Baltimore (*n* = 4)

^c^VIDUS study in Vancouver, BC (*n* = 3)

^d^ACCESS study in Vancouver, BC (*n* = 6)

^e^ALIVE study in Baltimore, MD (*n* = 2)

**Table 2:** Behavioral model of significant ^a^factors influencing ART adherence among people who inject drugs ^b^

Predisposing Factors	Enabling Factors	Need-Related Factors	Outcomes
*Socio-demographic barriers* ^c^ • Younger age [25–32, 34, 43, 53–55] • Female sex [25–29, 31, 33, 43, 53–55] • Low education [25, 27, 29, 32, 34, 47] • Minority (non-White) race/ethnicity [29, 33, 43, 47, 53] • Low health literacy [52] • Unemployment [33] *Substance use (SU) barriers* • Heroin use or injection [25, 27–30, 32, 43, 45, 53–55] Stimulant use or injection (alone or with opioids) [25, 27, 28, 30, 43, 47, 52–55] • Alcohol use [25, 28, 30, 31, 53, 51] • Binge drug use, relapse [29, 54] and overdose [25] • High addiction severity [45] and frequent SU [31] • SU to cope with stress *Social and structural vulnerability* • Homelessness [25, 28, 29, 53, 54] Incarceration [25, 29–31] • Sexual abuse history [26]	*Individual resources* • SU treatment use (medication-assisted therapies; inpatient and outpatient programs) [25, 27–29, 32–34, 43, 34, 47, 53–55] • Past ART experience [26, 28, 43, 51, 54] • Self-efficacy for ART use [47, 51] and disclosure [47] • Stable housing [29, 33, 34] • Health insurance [30, 34] • Empowerment [47, 51] • Social support [33] *Health service facilitators* • Good patient-provider relationships [30, 33, 34, 47] • Provider experience with HIV care [29, 53] • Accessible healthcare services [31, 34] • Directly administered ART (DAART) [50]	*Health status barriers* • Markers of poor physical health (detectable HIV viral load, low CD4 count,^c^ AIDS) [29–31, 33, 47, 53, 54] • Poor mental health (depression [33, 34, 45] especially in women [26, 47]) *Health beliefs and perceptions* • Perceived health status [47, 51] • ART-related beliefs [34, 47] • Sense of responsibility for protecting others [47] *Health risks* • Sex work [25, 29, 53, 54] • Risky sex partner(s) [26] • Syringe sharing [47] • Low self-efficacy for safe drug use [47]	*ART adherence measures* ^d^ • Pharmacy records [25, 27, 28, 29, 32, 43, 53–55] • Self-report [26, 30, 31, 34, 47, 50–52] • Biological markers [25, 30, 31, 33, 50] • Electronic monitoring [45]

^a^Significant factors defined as results that were statistically significant in univariate, bivariate, or multi-variate models at *p* < .10. Study references in bold indicate associations significant at *p* < .10 in final or multivariable models

^b^Adapted from the Gelberg-Andersen Behavioral Model for Vulnerable Populations. *Predisposing factors* are characteristics that could directly impact adherence, including socio-demographics, substance use barriers, and related sources of social and structural vulnerability. *Enabling factors* are resources that could facilitate utilization and be targeted by interventions, including individual resources and health service facilitators (e.g., characteristics of healthcare providers and services). *Need-related factors* include health status barriers, beliefs and perceptions, and health risks that could also influence adherence

^c^Directions of associations between CD4 count and adherence outcomes were mixed; unlike the studies referenced in the table, the following studies identified *high* CD4 count as a barrier to adherence: [27, 28, 32, 34, 43]

^d^Some studies examined multiple outcomes related to ART adherence. Pharmacy records included prescription refill data. Electronic monitoring was conducted via Medication Electronic Monitoring System (MEMS)

First, in the predisposing domain, studies provided evidence on socio-demographic barriers to ART adherence. Common demographic barriers were younger age (*n* = 13) and female sex (*n* = 12). Among younger PWID, financial barriers, low healthcare engagement, and high levels of risk taking and impulsivity may interfere with medication adherence [25]. Female PWID experience numerous vulnerabilities that have been shown to be associated with lower adherence, including sexual abuse and exploitation, trauma history, housing and financial instability (and engagement in transactional sex), and poor healthcare access [26, 27]. Other socio-demographic adherence barriers included low education (*n* = 6), minority (non-White) race/ethnicity (*n* = 5), low health literacy (*n* = 1) and unemployment (*n* = 1).

Also in the predisposing domain, various forms of substance use were associated with poor adherence across studies, including heroin (*n* = 11), stimulant (*n* = 10), and alcohol (n = 6) use. Binge drug use, relapse and overdose (*n* = 3), high addiction severity and frequent use (*n* = 2), and using substances to cope with stress (*n* = 1) were also associated with poor adherence. Related social and structural adherence barriers included homelessness (*n* = 5), incarceration (*n* = 4), and history of sexual abuse (*n* = 1). Homelessness and unstable housing carry particularly destabilizing effects on individuals’ daily routines, preventing privacy and safe medication storage, limiting contact with health services due to stigma, and further harming physical and mental health [28]. Similarly, incarceration interferes with adherence among PWID by disrupting treatment regimens and contact with providers [29–31].

Next, in the enabling domain, studies identified individual-level facilitators of ART adherence. In particular, engagement in substance use treatment, including medication-assisted therapies (MAT, especially methadone), supported ART adherence (*n* = 12). MAT involves frequent (often daily) contact with a system that may provide supportive counseling and referrals for medical and psychiatric conditions that challenge adherence. By reducing illicit drug use, MAT can also decrease the risk of incarceration, increase employment, and promote social relationships, all of which support adherence [32]. Psychosocial adherence enablers included self-efficacy for ART use (*n* = 3), empowerment (*n* = 2), and social support (*n* = 1), which have previously been shown to promote adherence in socioeconomically disadvantaged and marginalized substance using populations past ART experience (*n* = 5), stable housing (*n* = 3), and health insurance (*n* = 2) also enabled adherence.

Health service-related enablers of adherence included characteristics of strong patient-provider relationships (including trust of providers; *n* = 4) and provider experience in HIV care (*n* = 2). Good patient-provider relationships may depend on providers’ communication skills [33] and patients’ perceptions of shared decision making [34]. The accessibility of healthcare services also supported adherence (*n* = 2). Finally, directly administered ART (DAART) improved adherence among PWID in one intervention study.

Finally, in the need-related domain, poor physical health (as assessed by detectable HIV viral load and prior AIDS diagnosis, which could reflect poor control of HIV infection; *n* = 7) and mental health comorbidities (e.g., depression; *n* = 5) were associated with suboptimal adherence. The association between depression and mental health was strongest among women [26]. Health beliefs and perceptions, including poor perceived health status (*n* = 2), misperceptions about ART (e.g., believing that ART “eats” or interferes with methadone; *n* = 2), were negatively associated with adherence. Finally, specific health risk behaviors associated with poor adherence included engagement in sex work (*n* = 4), having risky sex partners (*n* = 1), sharing syringes (*n* = 1), and having low self-efficacy for safe drug use (*n* = 1). The competing priorities and demands posed by these health needs, which are persistent in many PWID populations, represent important challenges for adherence interventions.

## Discussion

Despite evidence that PrEP can help prevent HIV acquisition among high risk PWID [9], limited data suggest that PrEP adherence could be challenging for this population [11], Given the paucity of research on PrEP adherence among HIV-uninfected PWID (i.e., no published research studies outside of the Bangkok clinical trial and open-label study were available), we critically reviewed available evidence on ART adherence among HIV-infected PWID, a population that has been characterized by assumed and known challenges with accessing and using medications [35]. Drawing on the Behavioral Model of Healthcare Utilization for Vulnerable Populations [21], our review identified specific predisposing, enabling, and need-related influences on ART adherence among PWID that can inform PrEP adherence intervention research and development with this underserved population.

Specific factors that predispose certain subgroups of PWID to experience poor medication adherence may require specialized intervention content, delivery mechanisms, or outreach strategies. In particular, younger PWID and women had poor ART adherence in numerous studies. Younger PWID may have even more difficulty adhering to PrEP—a prevention tool—as evidence suggests that youth may struggle to prioritize the long-term health consequences of their behaviors and may benefit from positive, health-focused messaging (instead of risk-focused approaches) [36] and greater frequency of contact with providers [37]. Women who inject drugs often have an array of socio-economic vulnerabilities and histories of trauma and may benefit from PrEP interventions that provide strong linkages to sexual, reproductive, and mental health services [38]. Adapting interventions for the unique needs of these high risk subgroups of PWID who experience dual sexual- and drug-related HIV risks should be a priority within broader efforts to scale-up PrEP.

Distinct patterns of substance use interfered with adherence across studies included in our review, implying that PWID are not one homogenous group. As such, PrEP delivery to this population will require a thorough understanding of distinct drug usage patterns, related adherence challenges and intervention needs, and preferred clinical and community-based settings. For example, some subpopulations of PWID might prefer simple reminder systems that are inexpensive, easy to integrate into routine service delivery, and can have modest effects on adherence (e.g., daily phone calls, text messages, alarms, pill boxes). However, others may benefit from more intensive strategies (e.g., directly observed PrEP administration, perhaps coupled with MAT), which could be challenging in some settings. The unstable routines experienced by PWID also point to the need for working with counselors to anticipate changes in routines and keep medications accessible [39] or developing appropriate “cues” for pill taking [40]. Similarly, due to the high levels of homelessness and incarceration in this population, strategies such as case management should be considered within PrEP interventions for PWID [41].

Our review also identified enablers of adherence that could be leveraged in PrEP interventions for PWID. Studies provided strong evidence that substance use treatment (especially MAT) facilitates ART adherence. While drug treatment could be an important tool for promoting PrEP among some PWID, MAT may not be the most appropriate option for all PWID, especially those reporting stimulant and poly-substance use, for which effective medications are lacking, or alcohol use, which warrants additional research in relation to adherence in PWID [42]. Additional enabling factors identified in our review included self-efficacy for taking medications as prescribed. Recent evidence suggests that PrEP interventions should involve psychoeducational activities and motivational interviewing to boost self-efficacy. For example, the LifeSteps intervention, which involves enhanced counseling, problem solving techniques to improve self-efficacy, feedback on objective adherence measures, and reminder systems, has recently been adapted to improve PrEP adherence among high risk men who have sex with men [20]. Research is needed to determine if similar intervention strategies could be beneficial for PWID and whether peer-based models (e.g., patient navigation, support groups [43]) and provider-level interventions (e.g., to improve patient-centered interactions and willingness/ability to discuss PrEP [38]) could support PrEP adherence in PWID.

In the need-related domain, physical and mental health comorbidities, especially depression, interfered with adherence among PWID. PrEP interventions for this population will need to provide supported referrals for treatment and counseling [44]. Research should explore the feasibility, acceptability, and efficacy of case management, outreach services, coordination of health and social services, and integration of services (e.g., within primary care) [45]. Our review also underscored the need for interventions to address health risk factors common in this population (e.g., sharing syringes, [46] having risky sex partners, [26] and engaging in sex work, [27, 31]) because these factors not only interfere with adherence but also increase HIV risk (and thus the need for PrEP). One promising approach could involve integrating PrEP into low-barrier drug treatment programs or community-based harm reduction services (e.g., syringe exchange programs) that do not require abstinence from drug use and employ non-judgmental staff familiar with local PWID populations [47]. At a minimum, PrEP interventions for PWID should provide information and referrals to services for physical and mental health comorbidities, especially HCV, STI, and overdose prevention.

Three common limitations in the studies included in our review are limited generalizability, variability in outcome measurement, and observational designs. First, all studies had limited generalizability due to non-random and non-representative sampling, which is common with “hidden” populations with low service utilization and no established sampling frame [48]. A second limitation involves variation in how behavioral data were collected, and distinct biases for each method. While some studies used biological markers of adherence to ART [25, 30, 32, 33, 44, 49], many studies relied only self-reported medication-taking behaviors [26, 31, 34, 46, 50, 51], which could be subject to inaccurate recall, underreporting, and social desirability. However, self-report among PWID has been found to be reliable and valid compared to objective measures (e.g., biomarkers, administrative records) and repeated interviews [48]. Other studies relied on prescription refill data [27–29, 42, 52–54], which could overestimate actual medication taking [19, 28]. Third, studies included in our review were primarily observational and subject to unmeasured confounding; few intervention studies with randomized designs met our inclusion criteria [19, 50].

Beyond limitations and biases in the studies included in the systematic review, a limitation of the review itself results from potential differences between ART and PrEP adherence: both entail taking daily antiretroviral medications, but the motivations for adherence to each medication (as well as the conceptualization persistence) [15] of may differ. While ART must be taken consistently for life once a person is diagnosed with HIV in order to prevent the development of serious morbidity, PrEP adherence is predicated upon acknowledging risk behaviors and prevention-related motivation, and may only be necessary while an individual experiences high HIV risk [55]. Further studies will be needed to identify more nuanced differences and necessary adaptations of related intervention strategies. Additionally, limiting our review to U.S./Canadian settings where reduced generalizability and excluded studies in global settings; however, we determined that focusing on settings where PrEP was available was paramount to deriving useful implications for PrEP adherence-related research.

Despite the limitations of available research on ART adherence and the paucity of published data on PrEP adherence among PWID, lessons from our review yield several recommendations for research on interventions to improve PrEP adherence among PWID. In particular, innovative strategies may be needed to support adherence among highly vulnerable and marginalized sub-groups of PWID including younger PWID, women, and individuals experiencing homelessness and social and structural vulnerabilities. In-depth, formative research, including qualitative studies, should explore PrEP adherence challenges in this population. Finally, longitudinal studies involving innovative sampling and data collection methods are needed to monitor PrEP adherence among PWID and better understand the causal relationships between potential barriers described above and PrEP adherence in this population.

## Conclusions

In conclusion, PWID in many settings remain at high risk of HIV acquisition [1]. PrEP is a highly effective biomedical intervention for HIV prevention but systematic investigations of PrEP adherence among HIV-uninfected PWID exist. PrEP is currently approved as a single daily TDF/FTC pill, but other modalities are under study. Although new forms of PrEP (e.g., long-acting injectable PrEP, episodic PrEP) that do not require daily medication taking may be more acceptable among PWID, it will take time for these products to be tested and brought to market. In the meantime, assessing PrEP uptake in this marginalized and underserved population, and identifying related intervention needs, will be essential.

## Additional file

Additional file 1: ST1. Systematic review search terms used in electronic databases. (DOCX 18 kb)

## Abbreviations

ART: Antiretroviral therapy
DAART: Directly administered ART
HCV: Hepatitis C virus
MAT: Medication-assisted therapies
PrEP: Antiretroviral pre-exposure prophylaxis
PWID: People who inject drugs
