# Supplementary material for: Systematic review of HIV treatment adherence research among people who inject drugs in the United States and Canada: evidence to inform pre-exposure prophylaxis (PrEP) adherence interventions
Source: BMC Public Health. 2019 Jan 8;19:31. doi: 10.1186/s12889-018-6314-8 (PMC6323713; doi:10.1186/s12889-018-6314-8)
Supplement: Supplementary file 1 — ST1. Systematic review search terms used in electronic databases. (DOCX 18 kb) [file 12889_2018_6314_MOESM1_ESM.docx]

**Additional file**

**ST1. Systematic review search terms used in electronic databases**

| **Database** | **Search terms** |
| --- | --- |
| **PubMed** | ((("people who inject drugs" OR "injection drug use" OR "injection drug user" OR "injection drug users" OR "Substance Abuse, Intravenous"[Mesh] OR "intravenous drug use" OR "intravenous drug user" OR "intravenous drug users" OR "injection drug abuse" OR "injection drug abuser" OR "injection drug abusers" OR "intravenous drug abuse" OR "intravenous drug abuser" OR "intravenous drug abusers" OR “Needle sharing”[Mesh])) AND ("Pre-Exposure Prophylaxis"[Mesh] OR "pre-exposure prophylaxis" OR "PrEP" OR "HIV Infections/prevention and control"[Mesh] OR "Anti-HIV Agents"[Mesh] OR "Antiretroviral Therapy, Highly Active"[Mesh] OR “Emtricitabine, Tenofovir Disoproxil Fumarate Drug Combination”[Mesh] OR "Post-Exposure prophylaxis"[Mesh] OR "post-exposure prophylaxis" OR "HIV intervention" OR "antiretroviral therapy" OR "HIV prevention" OR "HIV control")) AND ("adherence" OR "compliance" OR "uptake" OR "retention") |
| **Web of Science** | TS="people who inject drugs" OR TS="injection drug user" OR TS="injection drug users" OR TS="injection drug use" OR TS="intravenous drug use" OR TS="intravenous drug user" OR TS="intravenous drug users" OR TS="intravenous drug abuse" OR TS="intravenous drug abusers" OR TS="intravenous drug abuser" OR TS="injection drug abuse" OR TS="injection drug abuser" OR TS="injection drug abusers" OR TS=”needle sharing”  AND  TS="pre-exposure prophylaxis" OR TS="PrEP" OR TS="HIV prevention" OR TS="HIV intervention" OR TS="HIV control" OR TS=”post-exposure prophylaxis” OR TS=”antiretroviral therapy”  AND  TS="adherence" OR TS="compliance" OR TS=”uptake” OR TS=”retention” |
| **EMBASE** | 'people who inject drugs' OR 'injection drug users' OR 'intravenous drug users' OR 'injection drug use' OR 'injection drug user' OR'intravenous drug use' OR 'intravenous drug user'/exp OR 'intravenous drug user' OR 'injection drug abuse' OR 'injection drug abuser' OR 'injection drug abusers' OR 'intravenous drug abuse'/exp OR 'intravenous drug abuse' OR 'intravenous drug abuser' OR'intravenous drug abusers' OR 'needle sharing'/exp OR 'needle sharing'  AND  'pre-exposure prophylaxis'/exp OR 'pre-exposure prophylaxis' OR 'prep' OR 'highly active antiretroviral therapy'/exp OR 'highly active antiretroviral therapy' OR 'antiretroviral therapy' OR 'emtricitabine plus tenofovir disoproxil'/exp OR 'emtricitabine plus tenofovir disoproxil' OR 'emtricitabine tenofovir disoproxil fumarate' OR 'harm reduction'/exp OR 'harm reduction' OR 'risk reduction'/exp OR 'risk reduction' OR 'hiv prevention' OR 'hiv intervention' OR 'hiv control' OR 'post exposure prophylaxis'/exp OR'post exposure prophylaxis' OR 'anti human immunodeficiency virus agent'/exp OR 'anti human immunodeficiency virus agent'  AND  'protocol compliance'/exp OR 'protocol compliance' OR 'patient compliance'/exp OR 'patient compliance' OR 'medication compliance'/exp OR 'medication compliance' OR 'drug uptake'/exp OR 'drug uptake' OR 'adherence' OR 'compliance'/exp OR'compliance' OR 'retention' OR 'uptake' |
| **PsycINFO** | ("people who inject drugs" OR "injection drug user" OR "injection drug users" OR "injection drug use" OR "intravenous drug use" OR "intravenous drug user" OR "intravenous drug users" OR "intravenous drug abuse" OR "intravenous drug abusers" OR "intravenous drug abuser" OR "injection drug abuse" OR "injection drug abuser" OR "injection drug abusers" OR ”needle sharing”) AND ("pre-exposure prophylaxis" OR "PrEP" OR "HIV prevention" OR "HIV intervention" OR "HIV control" OR ”post-exposure prophylaxis” OR ”antiretroviral therapy”)  AND  ("adherence" OR "compliance" OR ”uptake” OR ”retention”) |
